# Prions: Generation and Spread *Versus* Neurotoxicity

**DOI:** 10.1074/jbc.R114.568477

**Published:** 2014-05-23

**Authors:** Mark Halliday, Helois Radford, Giovanna R. Mallucci

**Affiliations:** From the Medical Research Council (MRC) Toxicology Unit, Hodgkin Building, University of Leicester, Lancaster Road, Leicester LE1 9HN, United Kingdom

**Keywords:** Alzheimer Disease, Gene Therapy, Neurodegeneration, Prion, Unfolded Protein Response (UPR), Alzheimer's, Neuroprotection

## Abstract

Neurodegenerative diseases are characterized by the aggregation of misfolded proteins in the brain. Among these disorders are the prion diseases, which are transmissible, and in which the misfolded proteins (“prions”) are also the infectious agent. Increasingly, it appears that misfolded proteins in Alzheimer and Parkinson diseases and the tauopathies also propagate in a “prion-like” manner. However, the association between prion formation, spread, and neurotoxicity is not clear. Recently, we showed that in prion disease, protein misfolding leads to neurodegeneration through dysregulation of generic proteostatic mechanisms, specifically, the unfolded protein response. Genetic and pharmacological manipulation of the unfolded protein response was neuroprotective despite continuing prion replication, hence dissociating this from neurotoxicity. The data have clear implications for treatment across the spectrum of these disorders, targeting pathogenic processes downstream of protein misfolding.

## Introduction

The “prion-like” nature of several neurodegenerative diseases has been proposed for a number of years. The central concept is the spread of self-propagating misfolded proteins from neuron to neuron throughout the brain, associated with more or less stereotypical patterns of neurodegeneration for specific diseases. Apart from prion protein (PrP)^2^ in the archetypal prion diseases (typified by Creutzfeldt-Jakob disease (CJD)), the evidence that amyloid-β1–42 (Aβ), Tau, and α-synuclein all propagate through the brain is compelling, with clear implications for the pathogenesis of Alzheimer disease, frontotemporal dementias and other tauopathies, and Parkinson disease. The spread (within the brain, at least) of neurodegenerative diseases through protein misfolding appears to be a truly generic phenomenon. However, the spread of misfolded protein is not evidence of neurodegeneration, and this raises critical questions about the link, or lack of it, between transmission of pathological proteins and their neurotoxic effects. What is the relationship between toxicity and “infectivity” in classic prion disease and other protein misfolding disorders? This review will consider this relationship, and discusses the advantages of focusing on neurotoxic pathways, downstream of the prion replication process, for treatment of this group of disorders.

## Prion Formation in Prion Disease

**FIGURE 1.**
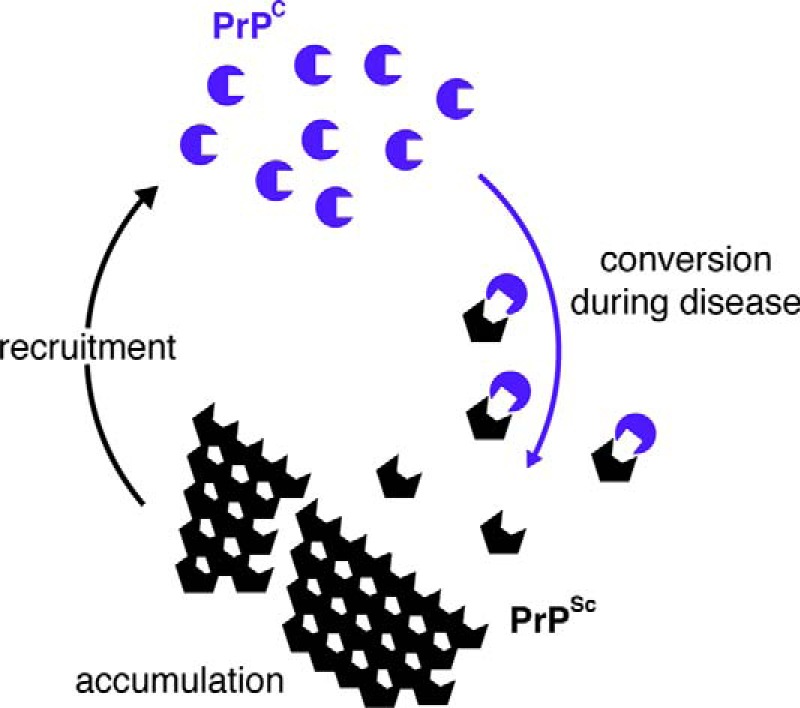
**Schematic of prion conversion.** Native prion protein (PrP^C^; *blue circular shapes*) is converted into PrP^Sc^ (*black hexagonal shapes*) in an autocatalytic process during prion replication. The two proteins have identical primary but different secondary structure. PrP^Sc^ is rich in β-sheet, is protease-resistant, and accumulates, recruiting more PrP^C^ for further cycles of conversion.

Prion diseases are fatal transmissible neurodegenerative disorders of humans and other mammals (see Watts and Prusiner (53) in this series). The classic veterinary disorder is scrapie in sheep; the most common human disease is CJD. The infectious agent is now widely accepted to be a protein that self-replicates, without the need for nucleic acids (1), confirming the “protein-only hypothesis” of transmission of these disorders first postulated by Griffith (2). The scrapie agent is a protein, present in aggregated form, highly insoluble in non-ionic detergents and partially protease-resistant, with a relative molecular mass of 27–30 kDa (3). Known as prion protein, PrP, it was found to be encoded by an endogenous gene, *PRNP* (4), which, intriguingly, was equally expressed in both infected and uninfected animals (4, 5). The normal product of the *PRNP* gene is PrP^C^, for cellular prion protein, a protease-sensitive protein of 33–35 kDa, whereas the previously isolated disease-specific protein was called PrP^Sc^, for scrapie-associated prion protein. These two isoforms of PrP share identical primary structures but differ in secondary and tertiary structure. The central mechanism of infectivity involves a change in the normal cellular isoform, PrP^C^, into PrP^Sc^ (6). This conversion is thought to be a post-translational change in conformation, which initiates the autocatalytic conversion of PrP^C^ into PrP^Sc^, by interaction with existing PrP^Sc^ molecules. As neurons are depleted of PrP^C^, newly synthesized PrP^C^ provides more substrate for conversion to PrP^Sc^, which accumulates, converting more PrP^C^, and so on (Fig. 1).

## Prion-like Spread in Other Neurodegenerative Diseases

The findings that Tau, Aβ, and α-synuclein are all capable of the type of templated conformational change that was first described for classic scrapie prions and that these changes could spread between cells were first established in cell models (7–9). Like classic prions, these proteins also form distinct conformers *in vivo,* and a number of elegant experiments confirmed that Aβ, mutant Tau, and mutant α-synuclein cause spread in regional pathology and disease progression in mouse models (10–13). More recently, the propagation and misfolding of wild type α-synuclein, giving rise to “sporadic”-type phenotypes in mice (12, 14), have been reported. Spread between animals has also been reported (see Holmes and Diamond (54) in this series), but transmission through repeated passage, as defines classic prion diseases, has not been seen.

Despite the universality of the prion-like spreading phenomenon, not all these models, including both classic CJD/scrapie and other protein folding diseases, show associated neurodegeneration, however. This raises the important concept that protein misfolding disorders have two aspects: first, within cell (*i.e.* cell-autonomous) processes that cause cellular dysfunction and ultimately neurodegeneration, and second, between cell (*i.e.* non-cell-autonomous) processes, through which pathology spreads. The link between the two is not clear, but it is important both for understanding disease mechanisms and for directing treatments.

## Dissociation of Prion Replication and Neurotoxicity

In the classic prion diseases, prion replication involves the conversion of native prion protein (PrP^C^) into the protease-resistant, disease-associated isoform that co-purifies with infectivity, PrP^Sc^ (see above). Given its accumulation in the brain and its capacity to transmit these fatal neurodegenerative conditions, PrP^Sc^ was generally assumed to be the neurotoxic species. However, the dissociation of toxic species (what kills neurons) and infectious agent (propagating prion protein) is now well established (15–17). Evidence for this dissociation appeared as early as 1993, with the landmark experiments of first Büeler *et al.* (18) and then Manson *et al.* (19), who showed that in the absence of PrP^C^, PrP^Sc^ was not toxic to brains of inoculated PrP-knock-out mice. Similarly, only wild type tissue expressing PrP^C^ grafted into the brains of PrP-null mice showed neurotoxic effects of prion infection (20). However, the key evidence came from the discovery of subclinical states of prion infection, characterized by experimental animals that were asymptomatic carriers of infectivity, never developing clinical disease throughout their lifespan (extensively reviewed by Hill and Collinge (16)). Similar subclinical states were observed by others (21–23), and the converse situation, neurodegeneration with minimal levels of PrP^Sc^, which was seen in certain inherited human prion diseases (24, 25) and in animal models (26), also supported the dissociation. Interestingly, switching off prion conversion in neurons during the course of prion infection, but allowing it to continue in astrocytes, leads to profound neuroprotection and rescue of neurons from prion toxicity despite massive extraneuronal accumulation of PrP^Sc^ (15, 27, 28). Removing the glycosylphosphatidylinositol anchor from PrP releases it from the neuronal cell surface and similarly prevents neurotoxicity (29) despite extensive extraneuronal PrP^Sc^ accumulation (this occurs over time despite low levels of expression of anchorless PrP in this model). Again, the findings discussed above support the idea that PrP^Sc^ itself is not directly toxic to neurons, but rather indicate that it is the process of prion conversion within them that leads to downstream (indirect) toxic effects. This is a critical finding as it implies the presence of generic, cellular pathways mediating toxicity in classic prion, and likely, in other neurodegenerative diseases.

This dissociation between prion propagation and neurotoxic effect is sometimes seen in the other neurodegenerative diseases. The landmark study by Clavaguera *et al.* (10), describing prion-like transmission of mutant human P301S Tau in mice, similarly showed spread of pathology, without neurodegeneration, as did the recent report of wild type α-synuclein (14). This is in contrast to the propagation and disease in other models (12, 13, 30). So cell-autonomous and non-cell-autonomous mechanisms co-exist, but they do not necessarily impinge equally on neurotoxicity in all cases. Both, however, clearly result from the same central phenomenon: the accumulation of misfolded proteins.

## Generic Mechanisms of Neurotoxicity

The prion-like neurodegenerative disorders, including the classic prion diseases, but also Alzheimer and Parkinson diseases and the tauopathies, as well as amyotrophic lateral sclerosis, all share the two key features: accumulation of misfolded proteins (irrespective of spread) and neuronal loss. We have used prion-infected mice to understand the link between protein misfolding and neurodegeneration. Prion-diseased mice are unique among mouse models of neurodegeneration as they truly recapitulate the human disorders and have extensive neuronal loss in association with accumulation of misfolded protein.

We studied tg37 mice used in our previous studies (15, 27, 28, 31–33). These mice overexpress PrP at around 3-fold wild type levels and succumb to Rocky Mountain Laboratory (RML) prion infection in around 12 weeks (31). Our first key observation biochemically was the finding that, in the context of increasing prion replication and rising levels of misfolded PrP, there was a sudden, abrupt reduction in the number of synaptic proteins at 9 weeks post infection (wpi). This correlated with critical reduction in both synapse number and neurotransmission and with accompanying behavioral decline and loss of object recognition memory. It was closely followed by neuronal loss, at 10 wpi (33). The reduction in synaptic protein levels at 9 wpi was clearly a catastrophic event, occurring at a critical moment during the disease process. We asked whether this drop reflected increased degradation of proteins or decreased synthesis. The ubiquitin proteasome pathway is known to be inhibited in prion disease, causing a reduction, not an increase, in protein degradation (34). We therefore asked whether protein synthesis was reduced through altered translational control mechanisms. Specifically, we examined the role of the unfolded protein response (UPR).

## The Unfolded Protein Response

The UPR is a protective cellular mechanism that is induced during periods of cellular and endoplasmic reticulum (ER) stress, which aims to maintain protein-folding homeostasis within the ER (35). The UPR has three main branches, all activated by rising levels of misfolded proteins in the ER. Two of these (the ATF6 and IRE1 branches) result in transcriptional changes that increase chaperone expression to enhance correct protein folding. The third, the PERK/eIF2α branch, results in a signaling cascade that leads to the transient shutdown of protein synthesis. Binding immunoglobulin protein (BiP) normally holds PERK in its inactive state, but when bound to unfolded proteins, it releases PERK, which autodimerizes and autophosphorylates. Phosphorylated PERK (PERK-P) phosphorylates eIF2α, which then inhibits the formation of the ternary complex that loads the 40 S ribosome onto the mRNA strand to be translated (36). Phosphorylated eIF2α (eIF2α-P) binds tightly to eIF2B, the guanine exchange factor that supplies the energy for the formation of the ternary complex, preventing it from supplying the GTP needed for loading to take place.

Thus, induction of the UPR leads to complex changes including the translation of molecular chaperones, the synthesis of lipids to increase ER volume, and a reduction in global protein synthesis to alleviate effects of overload of unfolded proteins inside the ER. UPR activation is usually a transient event; eIF2α-P is rapidly dephosphorylated by expression of the phosphatase GADD34/PP1, allowing normal protein translation to restart (37).

## The UPR in Prion Neurotoxicity

We analyzed activation of the UPR during rising levels of prion protein accumulation during the course of disease (see Fig. 3) as PrP is synthesized in the ER. We found that there was a progressive increase in PERK-P and eIF2α-P as the disease progressed (see Fig. 3*a*). GADD34 levels did not change, despite the rising eIF2α-P levels, suggesting that there was insufficient GADD34 to dephosphorylate the increased amounts of eIF2α-P. This shows that the PERK/eIF2α arm of the UPR is activated in prion disease, inhibiting protein translation and leading to a reduction in the levels of synaptic proteins. We also examined mice expressing even higher levels of PrP, with faster prion incubation times, and wild type mice. In each case, rising levels of misfolded prion protein triggered sustained activation of eIF2α-P and reduction in protein synthesis at a stage consistently ∼75% through the incubation period.

We measured total protein synthesis rates in the hippocampus via incorporation of radioactive methionine into protein in hippocampal slices, and also measured translation of specific mRNA by polysome profiling. A 50% decline in global protein synthesis was observed (see Fig. 3*b*), with a simultaneous reduction in the overall number of actively translating ribosomes at 9 wpi (33). Northern blots of SNAP-25 and β-actin mRNA also showed reduced active translation. In contrast ATF4 mRNA, which escapes eIF2α-P mediated inhibition of translation due to the presence of upstream open reading frames in its 5′-UTR (38), showed increased active translation. PrP mRNA did not show reduced translation, likely due to the presence of similar translational control elements within the PrP gene as ATF4.

## Therapeutic Manipulation of the UPR in Prion Neurodegeneration

**FIGURE 2.**
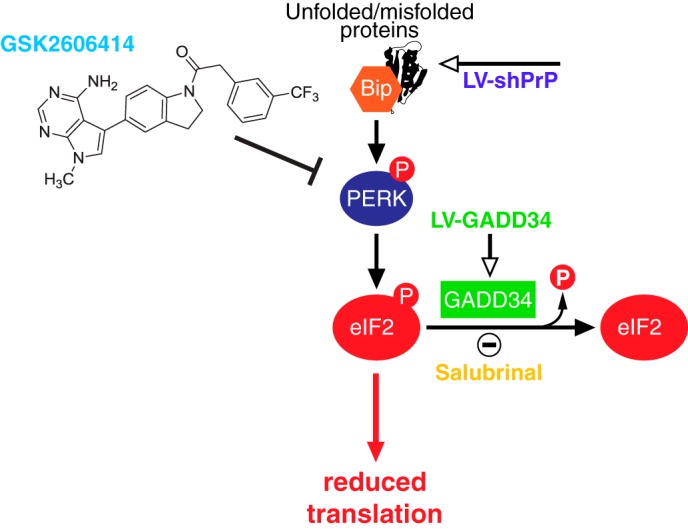
**Schematic representation of PERK branch of the UPR leading to translational repression and points of intervention.** Rising levels of misfolded proteins are detected by binding immunoglobulin protein (*BiP*) in the ER, activating PERK, which autophosphorylates, and in turn phosphorylates eIF2α, resulting in reduced translation. The decline in protein synthesis leads to the loss of key proteins, and hence synaptic failure and neurodegeneration. The points of action of GSK2606414, (a specific inhibitor of PERK), of lentivirus mediating RNAi of PrP (*LV-shPrP*), and of lentivirus overexpressing the eIF2α-P phosphatase, GADD34/PP1 (*LV-GADD34*) are shown. By inhibiting/preventing PERK phosphorylation (GSK2606414 and LV-shPrP) or dephosphorylating eIF2α-P (LV-GADD34), protein synthesis is restored. (Salubrinal prevents dephosphorylation of eIF2α-P, exacerbating the reduction of translation.)

Although transient eIF2α phosphorylation is beneficial to cells experiencing ER stress due to misfolded proteins, persistently high levels of eIF2α-P are likely to be detrimental. To test whether eIF2α-P is directly involved in prion neurodegeneration *in vivo*, we asked whether reducing the levels of eIF2α-P in prion disease would be neuroprotective. To do this, we overexpressed GADD34 using a lentiviral vector, to reduce eIF2α-P levels directly, and in parallel we used targeted RNAi of PrP to remove the source of UPR activation and prevent eIF2α-P formation (Fig. 2). We also asked whether increased levels of eIF2α-P exacerbate prion neurotoxicity by using salubrinal, an inhibitor of eIF2α-P dephosphorylation.

**FIGURE 3.**
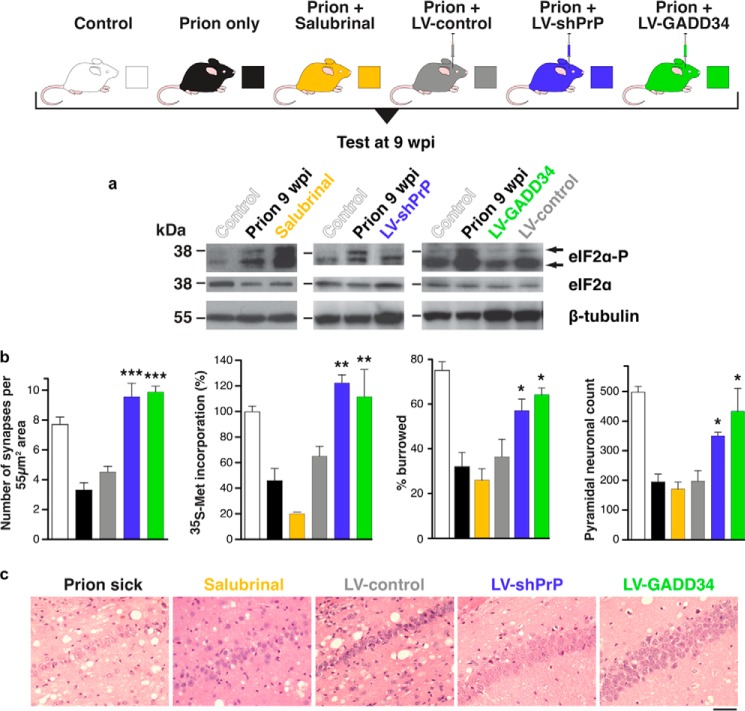
**Manipulation of the UPR rescues translation and is neuroprotective in prion-diseased mice.** *a*, lentivirally mediated RNAi against PrP (*blue bars*) or overexpression of GADD34 (*green*) reduces levels of eIF2α-P. *LV-shPrP*, lentivirus mediating RNAi of PrP; *LV-GADD34*, lentivirus overexpressing the eIF2α-P phosphatase, GADD34/PP1. *b*, restoring synapse number, global protein synthesis rates, burrowing behavior, and neuronal cell numbers when compared with untreated prion-diseased mice (*black*) or empty vector controls (*gray*). Salubrinal (*orange*) had a detrimental effect in the same experiments. All data in bar charts show mean ± S.E. *, *p* < 0.01; **, *p* < 0.001; ***, *p* < 0.005. *c*, neuroprotective effects of RNAi of PrP or GADD34 overexpression in CA1 pyramidal cell ribbon of hippocampus from prion-diseased mice. Adapted from Ref. 33.

At 9 wpi, mice injected with a lentivirus expressing GADD34 showed a similar level of PERK-P as untreated mice, demonstrating that the UPR was still being activated, but eIF2α-P levels were reduced (Fig. 3) (33). RNAi against PrP prevented the PrP-induced rise in PERK-P and eIF2α-P seen in untreated animals, confirming prevention of UPR activation. Both GADD34 overexpression and PrP knockdown restored global translation rates at 9 wpi. As a result, synaptic protein levels, synaptic transmission, and synapse number in prion-diseased mice treated with GADD34 or PrP knockdown were protected and equivalent to levels in uninfected control mice. Burrowing deficits were prevented, and there was extensive neuronal protection in the hippocampus, with no neuronal loss and markedly reduced spongiform change (Fig. 3). Importantly, targeted expression of GADD34 and focal PrP knockdown had a modest, but highly significant, effect on survival.

Critically, treatment with salubrinal had the opposite effect, by preventing dephosphorylation of eIF2α-P. Thus, eIF2α-P levels were markedly higher at 9 wpi than in prion-only controls, causing further repression of global translation. Salubrinal treatment resulted in earlier severe neuronal loss and significantly accelerated disease when compared with untreated prion-infected mice.

**FIGURE 4.**
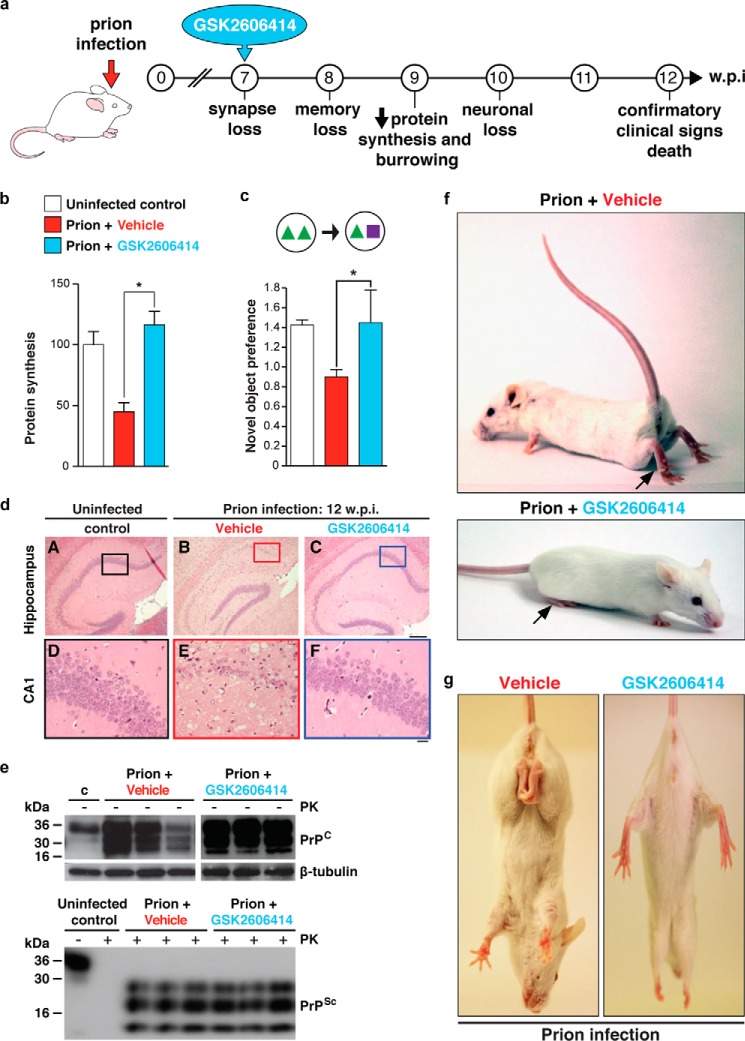
**PERK inhibition by GSK2606414 prevents clinical disease in prion-infected mice.** *a*, mice were treated with GSK2606414 (*blue*) or vehicle (*red*) from 7 wpi. *b–c*, GSK2606414 restored global protein synthesis rates (*b*), prevented loss of novel object memory (*c*), and afforded marked neuroprotection in hippocampus (*d*). *e*, levels of total PrP and PrP^Sc^ (as shown by proteinase K digestion (*PK*)) were unaffected by treatment. *f* and *g*, clinical cure in treated mice with normal posture and movement of hind legs. All data in bar charts show mean ± S.E. Controls represent mice inoculated with normal brain homogenate (*white bar*) (*n* = 12 for each) (*, *p* < 0.01). Adapted from Ref. 32.

The striking neuroprotection achieved by genetic manipulation of the UPR led us to predict that pharmacological inhibition of PERK/eIF2α-P would be similarly protective. We used a highly selective inhibitor of PERK GSK2606414 (39), originally designed as an anticancer compound (Fig. 2). We therefore treated prion-infected tg37 mice with GSK2606414, administered orally, from 7 weeks post infection. The PERK inhibitor prevented high levels of eIF2α-P and restored global protein synthesis rates. As with genetic manipulation of the UPR, the mice were clinically cured (32) and there was marked neuroprotection throughout the brain (Fig. 4), although effects on survival could not be assessed due to exocrine pancreatic toxicity associated with the compound, which resulted in weight loss necessitating termination of the experiment, despite the absence of prion clinical signs. The beneficial effects held true for animals treated both at the preclinical stage and also later in disease, when behavioral signs had emerged (32). Critically, the compound acts downstream, and independently, of the primary pathogenic process of prion replication and is effective despite continuing accumulation of PrP. Interestingly, we think the UPR is triggered by rising levels of total PrP synthesis in the ER rather than as a direct effect of aggregation of PrP^Sc^ as this occurs largely extracellularly, or within the endosomal compartment. We previously found that total PrP mRNA levels increase during prion infection, suggesting that increased synthesis of native PrP may cause misfolding and UPR activation (33), and there is evidence that overexpression of protein production can induce UPR markers (40).

## Wider Relevance of UPR Activation in Neurodegeneration: Restoring Global Protein Synthesis Is Good for Neurons

Increased levels of UPR activation and PERK-P and eIF2α-P have been described in the brains of Alzheimer disease, Parkinson disease, and prion disease patients (41–45), and genetic polymorphisms in PERK predispose to the tauopathy progressive supranuclear palsy (46). The significance of UPR overactivation is not clear, but several strands of evidence suggest that here too, promoting protein synthesis where this is chronically inhibited would be neuroprotective. Learning and memory depend on protein synthesis (47), and recent evidence has shown that inhibition of this pathway increases cognition in wild type mice (48) and prevents cognitive deficits in an Alzheimer disease mouse models (49). Restoring protein translation in a *Drosophila* and a mammalian neuronal cell model of amyotrophic lateral sclerosis using GSK2606414 has also shown benefits in reducing toxicity (50). The data therefore link this pathway with memory and cognition as well as with global neuronal health and viability in health and disease. The data support drug development programs targeting PERK and other members of this pathway for the treatment of prion, and potentially other UPR-inducing, neurodegenerative diseases such as Alzheimer and Parkinson diseases.

## Concluding Comments

The relationship between toxicity and infectivity in prion disease and other protein misfolding disorders is complex, and we have made our case for targeting the downstream effects of unfolded protein accumulation. However, intuitively, containing spread and reducing the stimulus to UPR induction must be beneficial, preventing the progression that characterizes the clinical evolution of these diseases as further brain regions are “recruited” over time. The question is how to do this. One approach is to administer disease-specific anti-misfolded-protein antibodies, which has been proposed for Tau and SOD1 (see Holmes and Diamond (54) review in this series). Alternatively, are there generic mechanisms of spread that could be targeted? It is clear that there are common structural features of oligomeric forms of these proteins; antibodies raised against oligomers of PrP also detect oligomeric forms of a number of other amyloid proteins, including Aβ (51). If such structural features were involved in a universal protein propagation mechanism, they could represent a common therapeutic target for many neurodegenerative diseases. Further, there may be generic cellular pathways, including exosomal and synaptic release mechanisms, underlying pathological spreading that could also potentially be targeted. Indeed, the neuroanatomical basis for this propagation was recently established by the demonstration of trans-synaptic spread of mutant Tau (39, 52), although exactly how this occurs is still unclear.

In summary, the discovery of generic phenomena, such as the spread of misfolded proteins and the effects of these on proteostasis, bring stimulating new insights into neurodegenerative diseases that may lead to new therapeutic approaches. However, we still need to understand much more both about these processes at a molecular level and about how cell-autonomous and non-cell-autonomous mechanisms relate to each other in these disorders before we can determine the balance needed when targeting these processes for therapy.

## Footnotes

2 The abbreviations used are:

PrP: prion protein
PrP^C^: cellular PrP
PrP^Sc^: misfolded PrP
CJD: Creutzfeldt-Jakob disease
Aβ: amyloid-β1–42
UPR: unfolded protein response
ER: endoplasmic reticulum
PERK: protein kinase RNA-like ER kinase
PERK-P: phosphorylated PERK
eIF2α-P: phosphorylated eIF2α
wpi: weeks post infection.
